# Special Issue: The Role of Microorganisms in the Evolution of Animals and Plants

**DOI:** 10.3390/microorganisms10020250

**Published:** 2022-01-23

**Authors:** Eugene Rosenberg, Ilana Zilber-Rosenberg

**Affiliations:** Department of Molecular Microbiology and Biotechnology, The George S. Wise Faculty of Life Sciences, Tel Aviv University, Tel Aviv 6997801, Israel; ilany43@gmail.com

It is now well established that all animals and plants harbor abundant and diverse microorganisms, including bacteria, archaea, viruses, and eukaryotic microorganisms. This conglomerate of the host and its microbes has been termed holobiont [1,2] and meta-organism [3]. During the last decade, the extent to which host–microbiome interactions affect the adaptation [4], behavior [5], development [6], and evolution [7] of animals and plants has become a major theoretical and experimental research topic. Consideration of the holobiont with its hologenome as a level of selection in evolution [8] has led to previously underappreciated modes of genetic variation and evolution [9]. In this Special Issue, we invited scientists to contribute articles on the different topics associated with the role of microorganisms in the evolution of plant and animal (including human) holobionts with the aim to improve understanding of how these complex systems adapt and evolve.

Nine interesting papers were included in this Special Issue, titled “Role of microorganisms in the evolution of animals and plants”. All of the articles discuss the interaction of microbiota and the host, as well as the importance of the wholeness of the structure termed holobiont. Three papers deal with plant microbiomes, three with animal microbiomes and three with more general aspects. Most of the articles lead to general concepts, such as tradeoffs between positive and negative effects of microbiota, potential application of microbiota in agriculture, how human intervention changed microbial evolution, and the vertical and horizontal transmission of microbiota.

Three articles deal with the role of microbiota in stress. The article by Slowinski et al. [10] argues that although symbiotic microbiota generally benefits their host, life history traits often demonstrate tradeoffs among one another. For example, they show that microbes in nematodes increase the host development rate but decrease host resistance to heat stress, suggesting that the complex interactions with microbiotas may mediate a tradeoff between host development and stress resistance. Furthermore, the authors suggest that such effects may depend on bacterially provided signals. Although the experimental research focused on a specific nematode, *Caenorhabditis elegans*, the concept of tradeoffs may be general.

The second paper on stress by Hernández et al. [11] discusses how a consideration of invasive plant species as holobionts improve our understanding of the mechanisms driving successful adaption and competitive advantage in new environmental conditions, such as salt stress. By studying a plant, *Elytrigia atherica*, which is invasive to salt marshes across the North Atlantic Coast from Northern Portugal to Southern Denmark, the authors show how microbiota influence the adaptability of their host plant to salt concentrations in soil. The data suggest that *E. atherica* is flexible in its association with soil bacteria and that ecotype-specific bacteria are dissimilar. This may help explain salinity and drought tolerance in relation to the local environmental needs of each ecotype.

The third article deals with temperature and antibiotic stress on the coral *Euphyllia paradivisa*, with and without photosymbionts. Meron et al. [12] demonstrate the significance of each component of the coral holobiont, the host, endosymbiotic algae, and bacteria, in responding to environmental stress. They observed a coherent link between the components of the coral holobiont, which affected the molecular and cellular processes of the coral, which in turn affected its fitness, especially under environmental stress conditions.

The report by Sheffer et al. [13] characterizes the total bacterial community of an-wasp spider, *Argiope bruennichi*, from geographically distant, but genetically similar, populations in Germany and Estonia. Overall, the microbiome differed significantly between populations and individuals, but not between tissue types. A novel bacterial symbiont, affiliated with Tenericutes, was found to be present and abundant in every tissue type in spiders from geographically distinct populations. Since it was also present in offspring, the authors conclude it is transmitted vertically. The data demands further inquest regarding the role of this novel but abundant symbiont in the ecology and evolution of its host Tenericutes spider.

Choosing the right mate can have a major impact on the fitness of a female’s offspring. Heys et al. [14] demonstrate that the microbiota in *Drosophila pseudoobscura* can affect host mate choice and that an intact microbiota is a key component of attractiveness in older males. The data indicate that the fly bacteria provides a signal used by females to assess male’s age, and that a damaged microbiome disrupts this signal. Thus, age-based preferences may break down in environments where the microbiome is impaired (temperature, antibiotics). For example, the authors report that *D. pseudoobscura* older males reared on an antibiotic-supplemented diet have decreased attractiveness to females.

Sariola and Gilbert [15] discuss a perspective on public health that places emphasis on microbial evolution through symbiotic associations between bacteria, viruses, and their eukaryotic hosts. They argue that, during the Anthropocene, human intervention has altered the conditions for microbial evolution in three ways. First, our changing relationship to microbes involves the Western world’s manufacturing an environment that is increasingly sterile and characterized by continually separating ourselves from nature. Second, the microbes, themselves, have changed during the Anthropocene. The unregulated use of antibiotics has created microbes that have evolved resistance to anti-microbial drugs. The third change that the authors discuss is the recent recognition of mutualistic symbiosis between humans and microbes. This article attempts to map out a holobiont perspective to public health.

Donald Smith and his coworkers at McGill University have contributed two papers to this Special Issue. One is a theoretical article, which discusses the critical role microbes have played in the origin and evolution of plants [16]. Starting with the uptake of cyanobacteria to form the first photosynthetic eukaryotic cell and continuing with acquisition of other microbes and microbial genes that have played a central role in plant nutrient acquisition, biotic and abiotic stress management, physiology regulation through microbe-to-plant signals, and growth regulation via the production of phytohormones. The authors argue that, by taking into account the history of plants and microorganisms, the influence of microbes on the evolution of plants is so profound that the concept of the plant is not viable without microbes. Thus, the holobiont concept should take greater precedence in plant sciences.

The second paper [17] discusses how the wide range of complex interactions between the phytomicrobiome and plants plays an important role in the evolution of plants and also in sustainable agriculture. This coevolution led to diverse beneficial and pathogenic interactions, as well as other diverse and abundant interactions. The authors compare microbes in the rhizosphere of agricultural plants to the well-studied mammalian gut microbiomes, as they are both essential for host survival and health. They propose that considering the plants and their phytomicrobiome as one unit is an essential step to sustainable agriculture, especially when compared to single-gene or multiple-gene improvements. The investigation of microbes isolated from unrelated plant species applied to established agricultural groups may lead to more robust plant holobionts.

The last article in the issue deals with the transmission of microbiota and their genes between generations [18]. After reviewing the various modes of vertical and horizontal transmission, the authors analyze published data on the fidelity of transmission. Arguments are put forth for both accurate vertical and horizontal transmission. Three underappreciated aspects of transmission are discussed. First, the transmission may be an essential function provided by the microbiome, not necessarily specific taxa. Second, horizontal transmission may be in many cases as accurate as vertical transmission. Third, because of the failure to detect rare species, reports of horizontal transmission may actually be the result of amplification of rare species. The fidelity of transmission provides a strong basis for each holobiont to be considered a unique biological entity and a level of selection in evolution, largely maintaining the uniqueness of the entity and conserving the species from one generation to the next.

## Data Availability

Not applicable.

## References

[1] Margulis L., Margulis L., Fester R. (1991). Symbiosis as a source of evolutionary innovation: Speciation and morphogenesis. Symbiogenesis and Symbionticism.

[2] Rohwer F., Seguritan V., Azam F., Knowlton N. (2002). Diversity and distribution of coral-associated bacteria. Mar. Ecol. Prog. Ser..

[3] Turnbaugh P.J., Ley R.E., Hamady M., Fraser-Liggett C.M., Knight R., Gordon J.I. (2007). The human microbiome project. Nature.

[4] Suárez J., Triviño V. (2020). What is a hologenomic adaptation? Emergent individuality and inter-identity in multispecies systems. Front. Psychol..

[5] Nagpal J., Cryan J.F. (2021). Host genetics, the microbiome & behaviour—A ‘Holobiont’ perspective. Cell Res..

[6] Simon J.C., Marchesi J.R., Mougel C., Selosse M.-A. (2019). Host-microbiota interactions: From holobiont theory to analysis. Microbiome.

[7] Rosenberg E., Zilber-Rosenberg I. (2018). The hologenome concept of evolution after 10 years. Microbiome.

[8] Roughgarden J., Gilbert S.F., Rosenberg E., Zilber-Rosenberg I., Lloyd E.A. (2018). Holobionts as units of selection and a model of their population dynamics and evolution. Biol. Theory.

[9] Zilber-Rosenberg I., Rosenberg E. (2021). Microbial-driven genetic variation in holobionts. FEMS Microbiol. Rev..

[10] Slowinski S., Ramirez I., Narayan V., Somayaji M., Para M., Pi S., Jadeja N., Karimzadegan S., Pees B., Shapira M. (2020). Interactions with a complex microbiota mediate a trade-off between the host development rate and heat stress resistance. Microorganisms.

[11] Hernández E.G., Baraza E., Smith C., Berg M.P., Salles J.F. (2020). Salt marsh elevation drives root microbial composition of the native invasive grass *Elytrigia atherica*. Microorganisms.

[12] Meron D., Maor-Landaw K., Eyal G., Elifantz H., Banin E., Yossi Loya Y., Levy O. (2020). The complexity of the holobiont in the Red Sea coral *Euphyllia paradivisa* under heat stress. Microorganisms.

[13] Sheffer M.M., Uhl G., Prost S., Lueders T., Urich T., Bengtsson M.M. (2020). Tissue- and population-level microbiome analysis of the wasp spider *Argiope bruennichi* identified a novel dominant bacterial symbiont. Microorganisms.

[14] Heys C., Lizé A., Lewis Z., Price T.A.R. (2020). *Drosophila* sexual attractiveness in older males is mediated by their microbiota. Microorganisms.

[15] Sariola S., Gilbert S.F. (2020). Toward a symbiotic perspective on public health: Recognizing the ambivalence of microbes in the Anthropocene. Microorganisms.

[16] Lyu D., Zajonc J., Pagé A., Tanney C.A.S., Shah A., Monjezi N., Msimbira L.A., Antar M., Nazari M., Backer R. (2021). Plant holobiont theory: The phytomicrobiome plays a central role in evolution and success. Microorganisms.

[17] Lyu D., Msimbira L.A., Nazari M., Antar M., Pagé A., Shah A., Monjezi N., Zajonc J., Tanney C.A.S., Backer R. (2021). The coevolution of plants and microbes underpins sustainable agriculture. Microorganisms.

[18] Rosenberg E., Zilber-Rosenberg I. (2022). Reconstitution and transmission of gut microbiomes and their genes between generations. Microorganisms.

